# Supplementary material for: Demonstration project of oral TDF-containing PrEP, administered, once-daily orally to men having sex with men (MSM) and transgender women (TGW) in India: Study protocol
Source: PLoS One. 2023 Jun 23;18(6):e0287454. doi: 10.1371/journal.pone.0287454 (PMC10289442; doi:10.1371/journal.pone.0287454)
Supplement: Attachment — Submitted filename: Review.pdf [file pone.0287454.s001.pdf]

- There are many instances throughout the manuscript where data is provided, or assertions are made based upon prior research where the source(s) are not cited or are cited incorrectly. For example, the statement in lines 47-48: “India has demonstrated considerable success with a 37% decline in new HIV infections since the year 2010, yet around 69,000 new infections occurred in 2019” and the statement in lines 97-99: “a gap between efficacy and effectiveness is predicted as many clinical trial researchers cited adherence as a major challenge for efficacy” have no sources citated. For the statement in lines 392-394: “National AIDS Control Organization has played a pivotal role in the prevention and control of HIV/AIDS seen as a reduction in prevalence to 0.22%” the source is incorrectly attributed to reference number 3 (The NACO 2014-2015 Annual Report – national HIV prevalence in India estimates at 0.35% in this report) when it is actually from reference number 1 (the NACO and ICMR-National Institute of Medical Statistics India HIV Estimates 2019 Report). These errors throughout the manuscript require correction.

- The study purpose/objectives/aims, where described at different points in the manuscript are inconsistent. You should use the WHO “PrEP demonstration projects. A framework for country level protocol development” as a guide, and focus on describing the overarching purpose of the study - i.e. to evaluate the feasibility and potential impact of implementing HIV PrEP as part of combination prevention for HIV among MSM and TGW in India. Then describe the factors you will assess to evaluate that main purpose - i.e. by assessing adherence to PrEP and the incidence of HIV infection among study participants compared to the incidence of HIV among a similar population in the same area. You can then further elaborate on specific indicators/measures related to adherence such as facilitators/barriers to adherence, willingness to pay for PrEP, retention in the study, etc. as well as your secondary outcomes of interest that you plan to assess in this study.

- There are multiple instances throughout the manuscript where the full word/phrase/term that an abbreviation represents does not precede the first use of that abbreviation that should be corrected. This should be done for the first use of an abbreviation in the abstract as well as its first use in the body of the manuscript. For example, human immunodeficiency virus should precede “(HIV)” on the first use of the abbreviation in the abstract in line 17, and again on its first use in the body of the manuscript in line 44.

- Highly recommend scientific copy editing of the manuscript to improve readability, comprehension, and correct grammatical errors

- Line 17: the full word/phrase/term that an abbreviation (HIV in this case) represents should be included prior to that abbreviation first use in the text

- Line 18: should include full term for “MSM” before the abbreviation

- Line 23: the full word/phrase/term that an abbreviation (TDF in this case) represents should be included prior to that abbreviation first use in the text

- Lines 29-30: you describe your primary outcomes of interest here but do not include “the proportion of PrEP users who tests HIV positive during the study period” which is listed as a primary outcome later in the manuscript (line 271). You also describe “sexual behavioral changes with PrEP use” as a primary outcome of interest here, but this is not listed as a primary or specifically as a secondary outcome elsewhere in the manuscript.

- As this study attempting to assess the feasibility of PrEP in the populations at highest risk of HIV infection in India, which you postulate are the MSM and TGW populations, you should include data on the incidence of HIV infection in these populations in the background section that supports that these populations are in fact the highest risk populations in India for new HIV infection. For example, define how many of the approximately 69,000 new HIV infections in line 48 occurred amongst those that identify as MSM or TGW.

- Lines 52-57: this data describing increased likelihood for HIV infection among the MSM population does not contribute much to the rational for this study – you aren't targeting any of these specific sub-populations of MSM

- Line 66: while there is data to support male circumcision as effective in the prevention of HIV among heterosexual men, this data does not support prevention solely from insertive vaginal intercourse (the studies didn't obtain any data on the specific types of sexually activity with the participants' partners)

- Lines 68-69: while VMMC may not be culturally acceptable in India, this is irrelevant as there is no evidence that VMMC is effective in the prevention of HIV infection among your target population (MSM and TGW), so this isn't good justification to pursue alternative prevention strategies in your context

- Lines 72-74: per the most recent update of the clinical practice guideline for PrEP for the prevention of HIV in the US, the US CDC recommends offering PrEP to adults and adolescents whose sexual activity or injection drug use practices place them at substantial ongoing risk of HIV acquisition

- Lines 74-78: given the updated 2015 PrEP recommendations from the WHO which includes using PrEP as part of a combination prevention package, the 2014 recommendation is rather irrelevant

- Line 96: UNAIDS recommendations were not discussed previously, nor otherwise referenced, so it is unclear why they are mentioned here

- Line 102: should specify that the "real world" setting in this case is in India

- Lines 132-140: you need to provide a description of the community-based setting (none included) and more detail on the facility-based setting such as specific location/facility, why that location was chosen, if it is embedded in an existing clinic (and if so, what type of clinic/other services offered) or was created for the sole purpose of this trial

- Lines 133-134: why did you select these states for your study sites? Are these the states with the highest HIV prevalence (although incidence would be a more appropriate measure for this study) in India and is this the most recently available data? You report that the HSS is conducted yearly earlier in the background section.

- Lines 135-136: what is the reason that you decided to establish the community-based vs the facility-based setting in one location vs the other? This should be discussed.

- Lines 137-138: Why do you expect differences in stigma in between the two locations based on geographic location? This should be discussed.

- Lines 149-150: how did you determine the need for/sufficiency of 20% of the study population being TGW and how do you plan to ensure that 20% of your participants will be TGW? This should be discussed.

- Lines 154-161: the wording here suggests that your criteria defining substantial risk for HIV infection are from the WHO PrEP demonstration projects guidelines – they are not as this guideline does not specify any specific criteria to define substantial ongoing risk for any population. You should specify why you chose these specific criteria to identify participants at substantial ongoing risk for HIV infection and/or what guideline/reference you obtained them from. Why are you not including persons with an HIV positive sex partner, persons using injection drugs whose practices place them at substantial ongoing risk of HIV acquisition?

- Line 161: the full word/phrase/term that an abbreviation (HBV and HCV in this case) represents should be included prior to that abbreviation first use in the text

- Lines 161-162: what are the contraindications to oral TDF containing PrEP medications used in your study? This should be discussed.

- Line 175: the full word/phrase/term that an abbreviation (CBOs/NGOs in this case) represents should be included prior to that abbreviation first use in the text

- Lines 187-195: you have not included any measures to assess participants for acute HIV infection during screening such as screening for signs or symptoms suggestive of acute HIV infection within the previous four weeks or high-risk exposure within four weeks of starting PrEP. This is an extremely important step in the assessment of patients for initiation of PrEP to prevent starting PrEP in someone with acute HIV infection.

- Lines 188-195: the specific tests to be used in this HIV screening process for your study need to be described i.e., manufacturer and name of test along with what type of test it is such as "the *Abbot DETERMINE™ HIV-1/2 AG/AB COMBO*, a 4th generation HIV antigen/antibody test that detects both HIV-1/2 antibodies and free HIV-1 p24 antigen"

- Line 196: Within what timeframe will participants undergo the enrollment visit after screening? This should be discussed.

- Lines 203-204: recommend removing "and" from "All enrolled and HBV-non immune" to clarify that it will be the HBV non-immune participants enrolled in the study who will be offered HBV vaccination.

- Lines 206-219: frequency of HIV screening, serum creatinine measurement (including any plans for increased frequency of measurement for those with risk factors for renal disease such as hypertension, diabetes, etc.), and questionnaires/surveys to obtain data on sexual behaviors, risk perception, facilitators/barriers to adherence, use of other HIV prevention options, behavioral/psychologic health to measure your study objectives should be discussed here. Screening for symptoms suggestive of acute HIV infection should also be included at study visits.

- Lines 208-210: as written it seems that side effects will be assessed, and adherence counseling will be done only during the first 6 visits – this should be done at every study visit

- Lines 210-211: you need to describe how these samples will be managed/stored for future tenofovir drug level monitoring as well as the specific test you will use to test tenofovir drug levels.

- Lines 216-218: why are you not including *C. trachomatis* and *N. gonorrhea* PCR testing on pharyngeal and rectal swab samples to screen for pharyngeal/rectal gonorrhea and rectal chlamydia?

- Lines 218-219: there is generally no role for serologic screening for HSV in asymptomatic persons as HSV-2 serologic tests have a low specificity and a high false-positive rate when used for screening asymptomatic individuals, and there is no specific treatment warranted for an asymptomatic individual. What is your rationale for including screening for HSV in your study?

- Line 219: what specific type of HSV testing will be performed for a participant with an ulcer suspected to be caused by HSV? This should be discussed.

- Lines 223-224: it would be ideal to include this in determining eligibility to help minimize study drop-outs

- Lines 235-240: how many days supply of PrEP will participants receive and at what visits will they receive it? This should be discussed.

- Lines 235-240: you discuss elsewhere in the manuscript that assessing participants willingness to pay for PrEP, the willingness of study participants to use PrEP if available at a subsidized cost under the program, and the median cost of PrEP participants are willing to pay as objectives of the study. However, there is no discussion here (or elsewhere) regarding participants paying (or not paying) for the PrEP as provided in the study or the subsidization of the cost of PrEP to the participants by the study. This should be discussed.

- Line 238: You should specify the dose of emtricitabine in each tablet.

- Lines 237-238: you state here that participants will receive specifically Tenofovir Disoproxil Fumarate-Emtricitabine for PrEP as the study medication, but elsewhere throughout the manuscript you refer to/imply that either Tenofovir Disoproxil Fumarate-Emtricitabine or Tenofovir Disoproxil Fumarate-Lamivudine may be used in the study (lines 162, 167). If participants will be receiving only Tenofovir Disoproxil Fumarate- Emtricitabine as the study medication for PrEP in this study, then references to Tenofovir Disoproxil Fumarate-Lamivudine should not be included other than for background information.

- Lines 258-261: your method for determining acceptability is unclear as written. Are you defining acceptability as the proportion of persons who actually enroll in the study and initiate PrEP out of all the persons determined to be eligible and offered enrollment in the study.

- Lines 266-270: again, it is unclear how you are defining/determining acceptance based on this description.

- Line 280: how are you determining the number of unprotected sexual acts in the last three months (you provide no description anywhere in the manuscript of a study questionnaire/survey to obtain this data at follow-up visits)? Is this the sole measure for your previously stated study objective of evaluating change in sexual behaviors in response to PrEP? Number, type, and new sex partners as well as type of sex acts would provide additional valuable insight regarding changes in sexual behavior.

- Lines 281-282: how are you determining the number of study participants exhibiting anxiety, depression, and suicidal ideation (you provide no description anywhere in the manuscript of a study questionnaire/survey to obtain this data at follow-up visits)?

- Lines 283-284: how are you determining the proportion of PrEP users willing for paid PrEP (you provide no description anywhere in the manuscript of a study questionnaire/survey to obtain this data at follow-up visits)?

- Lines 285-286: how are you determining the median cost of PrEP, participants willing to pay (you provide no description anywhere in the manuscript of a study questionnaire/survey to obtain this data at follow-up visits)?

- Line 287: how are you determining the reasons for choosing or declining PrEP (you provide no description anywhere in the manuscript on how this data will be obtained when eligible participants are offered enrollment or a study questionnaire/survey to obtain this data at follow-up visits)?

- Line 288: how will you determine the number of successful partner referrals (you provide no description anywhere in the manuscript on how this data will be obtained/tracked)?

- Lines 291-292: here you state "Serious adverse events are those that are considered life-threatening", but later in lines 307-310, you describe multiple different scenarios that define a serious adverse event. This discrepancy needs corrected.

- Lines 355-368: what are the statistical tests you will use to perform these analyses? This should be discussed.

- Lines 368-370: you state that no baseline data for HIV incidence among MSM in India is available, but the "National AIDS Control Organization & ICMR-National Institute of Medical Statistics. India HIV Estimates 2019", which you cite in your manuscript, contains data on HIV incidence among MSM. Comparing the HIV incidence among participants in your study to the incidence of HIV among a similar population in the same location would be very informative to determine the potential impact of an oral PrEP program on the prevention of HIV and is a primary outcome of interest suggested by the WHO "PrEP demonstration projects. A framework for country level protocol development".

- Lines 371-374: why are you not comparing the two service delivery models?

- Line 411: multi-center is not an appropriate description of this study as your two sites have different service delivery methods (community-based vs facility-based).

- Lines 468-470: how does excluding persons with HCV infection prevent behavioral disinhibition among PrEP participants and prevent any future HCV positivity in this study?

- Figure 1: includes an option for persons determined non-eligible for the study to be deferred and re-screened. This is not discussed/further described at any point in description of the screening/enrollment process in the manuscript. Is this actually part of the study implementation? If so, this needs to be described in the manuscript.

- Figure 2: should be designated as a table.

- Figure 2: I'm assuming "Data Collection" is referring to completion of a study case report form/questionnaire/survey – this event should be titled as such. "Data Collection" is extremely vague.

- Figure 2: genital/anal swab collection for NG/CT testing was not discussed in the manuscript, only urine PCR testing for NG/CT was discussed. Why are you not including pharyngeal swabs for NG/CT PCR testing?

- Figure 2: you list "HBsAG testing" at screening, but not hepatitis B surface antibody or hepatitis B core antibody testing. Without these tests, how do you plan to identify HBV non-immune participants to offer HBV vaccination? Additionally, if you plan to offer vaccination to participants who are HBV non-immune, why are you testing for HBsAg again at the 6- and 9-month visits?

- Figure 2: What is the purpose of the "Storage of 2ml plasma sample" event? Is this for the testing of TDF levels? If so, this should be specified. Additionally, in the "Study Visits" section of the manuscript, lines 210-211, you state that "The blood sample will be collected at each visit for future Tenofovir level monitoring" but per the SOE in this figure, it is only collected at enrollment (which doesn't make sense as the participant will not have been taking PrEP at this point), 3-month, 6-month, 9-month, and 12-month visits. This discrepancy needs to be corrected.
